# Optimization of *Plasmodium vivax* sporozoite production from *Anopheles stephensi* in South West India

**DOI:** 10.1186/s12936-021-03767-2

**Published:** 2021-05-18

**Authors:** Ajeet Kumar Mohanty, Charles de Souza, Deepika Harjai, Prathamesh Ghavanalkar, Mezia Fernandes, Anvily Almeida, Jayashri Walke, Suresh Kumar Manoharan, Ligia Pereira, Rashmi Dash, Anjali Mascarenhas, Edwin Gomes, Thanyapit Thita, Laura Chery, Anupkumar R. Anvikar, Ashwani Kumar, Neena Valecha, Pradipsinh K. Rathod, Rapatbhorn Patrapuvich

**Affiliations:** 1 Field Unit, National Institute of Malaria Research, Campal, Goa 403001 India; 2 Goa Medical College and Hospital, Bambolim, Goa 403202 India; 3 Department of Chemistry, University of Washington, Seattle, WA 98195 USA; 4 Drug Research Unit for Malaria (DRUM), Center of Excellence in Malaria Research, Faculty of Tropical Medicine, Mahidol University, Bangkok, 10400 Thailand; 5 National Institute of Malaria Research (ICMR), Sector 8, Dwarka, New Delhi 110077 India; 6 Present Address: ICMR-Vector Control Research Centre, Medical Complex, VCRC Road, Indra Nagar, Priyadarshini Nagar, Puducherry, 605006 India

**Keywords:** *Anopheles stephensi*, Insectary, *Plasmodium vivax*, Membrane-feeding assays, Sporozoite

## Abstract

**Background:**

Efforts to study the biology of *Plasmodium vivax* liver stages, particularly the latent hypnozoites, have been hampered by the limited availability of *P. vivax* sporozoites. *Anopheles stephensi* is a major urban malaria vector in Goa and elsewhere in South Asia. Using *P. vivax* patient blood samples, a series of standard membrane-feeding experiments were performed with *An. stephensi* under the US NIH International Center of Excellence for Malaria Research (ICEMR) for Malaria Evolution in South Asia (MESA). The goal was to understand the dynamics of parasite development in mosquitoes as well as the production of *P. vivax* sporozoites. To obtain a robust supply of *P. vivax* sporozoites, mosquito-rearing and mosquito membrane-feeding techniques were optimized, which are described here.

**Methods:**

Membrane-feeding experiments were conducted using both wild and laboratory-colonized *An. stephensi* mosquitoes and patient-derived *P. vivax* collected at the Goa Medical College and Hospital. Parasite development to midgut oocysts and salivary gland sporozoites was assessed on days 7 and 14 post-feeding, respectively. The optimal conditions for mosquito rearing and feeding were evaluated to produce high-quality mosquitoes and to yield a high sporozoite rate, respectively.

**Results:**

Laboratory-colonized mosquitoes could be starved for a shorter time before successful blood feeding compared with wild-caught mosquitoes. Optimizing the mosquito-rearing methods significantly increased mosquito survival. For mosquito feeding, replacing patient plasma with naïve serum increased sporozoite production > two-fold. With these changes, the sporozoite infection rate was high (> 85%) and resulted in an average of ~ 22,000 sporozoites per mosquito. Some mosquitoes reached up to 73,000 sporozoites. Sporozoite production could not be predicted from gametocyte density but could be predicted by measuring oocyst infection and oocyst load.

**Conclusions:**

Optimized conditions for the production of high-quality *P. vivax* sporozoite-infected *An. stephensi* were established at a field site in South West India. This report describes techniques for producing a ready resource of *P. vivax* sporozoites. The improved protocols can help in future research on the biology of *P. vivax* liver stages, including hypnozoites, in India, as well as the development of anti-relapse interventions for vivax malaria.

**Supplementary Information:**

The online version contains supplementary material available at 10.1186/s12936-021-03767-2.

## Background

Malaria is a disease caused by infection with *Plasmodium* parasites, which are transmitted to humans through the bites of infected female *Anopheles* mosquitoes. In 2019, malaria parasites infected more than 229 million people globally and caused an estimated 409,000 deaths [1]. Of the five *Plasmodium* species that infect humans, *Plasmodium vivax* is the most prevalent, with almost half of the world’s population at risk of infection. A recent 2019 report used high-resolution maps of global *P. vivax* burden during 2000–2017 to estimate 14.3 million *P. vivax* cases in 2017 [2]. Approximately 82% (11.7 million cases) of these were in high-burden countries: India, Pakistan and Ethiopia, of which the majority were in India [2]. The burden of *P. vivax* in India is very complex, in part, because of the highly variable eco-epidemiology in different regions and different people, and the presence of multiple anopheline vectors. Vivax disease presentations can vary substantially, not only due to multiple relapse patterns with varying latency periods but also resistance to control measures, particularly in urban areas [3]. Indian *P. vivax* populations also have high genetic variability [4]. All this, together with the limited knowledge of the biology of the latent liver-stage hypnozoites, presents challenges for *P. vivax* control efforts and ultimately, impedes the goal of malaria elimination in India.

Reliable access to *P*. *vivax* sporozoites remains a bottleneck to the advancement of research on liver-stage and hypnozoite biology, which in turn are critical for both drug discovery and vaccine development. Continuous culture techniques for *P. vivax* parasites are lacking. To obtain *P. vivax* sporozoites, female *Anopheles* mosquitoes have to be fed on patient-derived blood using a standard membrane-feeding technique [5]. Hence, mosquito insectaries for studying *P. vivax* development are generally located close to patient blood collections at clinical sites and laboratories conducting liver-stage studies [6]. Indeed, mosquito membrane-feeding laboratories have been established in several endemic regions with the aim of producing *P. vivax*-infected mosquitoes with infective sporozoites for liver-stage research [7–9]. These include the use of *Anopheles dirus* [7] and *Anopheles cracens* [8], and *Anopheles darlingi* [9], the main malaria vectors in Southeast Asia and Amazonia, respectively. The overall mean sporozoite yield per mosquito obtained for the Southeast Asia vectors, *An. dirus* (62,514; range 202–233,168) [7] and *An. cracens* (26,112; range 323–79,310) [8] compares favourably with that observed for *An. darlingi* maintained in Peru (6539; range 57–98,600) [9].

The US NIH-sponsored Malaria Evolution in South Asia (MESA) programme project, an International Center of Excellence for Malaria Research (ICEMR), has established an insectary at the National Institute of Malaria Research (NIMR) field unit in Goa, India. The unit maintains stocks of *Anopheles stephensi*, which is a major urban malaria vector in India and particularly abundant in Goa [10]. Taking advantage of routine access to patient-derived blood samples from Goa Medical College Hospital, experimental *P. vivax* infections of *An. stephensi* have been conducted by the MESA programme to understand the dynamics of parasite development in mosquitoes, parasite-mosquito interactions, and the production of *P. vivax* sporozoites [11, 12]. Although wild-caught *An. stephensi* are more efficient at providing *P. vivax* sporozoites than laboratory-colonized populations [12], batch-to-batch variations in mosquito infectivity limit the reproducibility of the system. The present study aimed to improve the robustness of *P. vivax* sporozoite production in *An. stephensi.* The conditions for rearing high-quality *P. vivax*-infected mosquitoes were improved, and membrane-feeding procedures were optimized to increase the routine supply of *P. vivax* sporozoites.

## Methods

### Ethics approval

All approvals for collecting blood from malaria patients and conducting the study were obtained from the Institutional Ethics Committee of ICMR-National Institute of Malaria Research, New Delhi (ECR/NIMR/EC/2017/44), Goa Medical College and Hospital (GMC), the University of Washington Institutional Review Board, NIH/NIAID Division of Microbiology and Infectious Disease (DMID), Health Ministry Screening Committee (HMSC) of the Government of India and by the Government of Goa Public Health Department.

### Mosquito rearing

The maintenance of mosquito colonies was carried out at the secure insectary of the MESA-ICEMR laboratory of the NIMR field station in Goa, India. The larvae were reared in plastic trays (25 × 30 × 4 cm) containing 1.5 L of reverse osmosis water, and maintained in the laboratory at 27 ± 2 °C and a relative humidity of 70 ± 5% with alternating 12 h cycles of light and dark. The larvae were fed daily with a pinch of powdered Tetramin fish food (TETRA GMBH, Melle, Germany) until pupation. The pupae were collected from the trays and transferred to plastic bowls containing 200 mL of reverse osmosis water and kept inside a closed cage until the adults emerged. The adult mosquitoes were continuously fed with cotton pads soaked in 5% (w/v) glucose solution mixed with 5% (v/v) multivitamin syrup solution (Haemo-Vit, Boss Pharmacare Co. Ltd, Samut Sakhon, Thailand). A detailed protocol is presented in the supplementary information (Additional file 1: File S1).

### *Plasmodium vivax*-infected blood samples

*Plasmodium vivax*-infected blood samples from 38 patient volunteers were collected locally at Goa Medical College and Hospital (GMC). *Plasmodium* infection was confirmed by microscopic analysis of Giemsa-stained thin blood smears. A detailed description of the enrolment criteria and sample processing protocols has been published elsewhere [13]. Patients were informed about the study by the project staff, and informed consent was obtained from each participant prior to blood collection. After collecting venous patient blood (6 mL) in an acid citrate dextrose vacutainer (BD, India), the samples were placed in a 37 °C thermos flask to prevent gametocyte exflagellation and immediately transported from GMC to the MESA-ICEMR insectary. Because the GMC blood collection site is about 7 km from the insectary, blood with *P. vivax* could be fed to the mosquitoes through a membrane feeder within 1 h after being drawn from patients.

### Mosquito infection by membrane feeding

The wild-caught and laboratory-established *An. stephensi* mosquitoes were reared and maintained at the MESA-ICEMR insectary as previously described [12]. The wild mosquito larvae and pupae were collected from the curing waters of natural breeding habitats in construction sites in the city of Ponda, Goa. Five- to 6-days-old adult females of wild *An. stephensi* and laboratory-colonized *An. stephensi* in their 66^–^86th generation were used for membrane-feeding experiments as described in earlier reports [11, 12]. To confirm mono *P. vivax* infection of blood samples, an additional rapid diagnostic test (RDT) (FalciVax, Zephyr Biomedicals) was performed at the insectary prior to mosquito feeding. For each 3 mL blood feeding, mosquitoes (100 per cup) were starved (overnight for the wild colony and 4–6 h for the laboratory colony) and allowed to membrane feed for 60 min in a temperature-controlled room at 27 ± 2 °C and a relative humidity of 75 ± 2%. The numbers of engorged mosquitoes were recorded and unfed or partially fed mosquitoes were removed. The blood-feeding rate was recorded as the number of engorged mosquitoes/number of mosquitoes tested × 100 [14]. The engorged mosquitoes were kept in plastic cups in a Percival incubator maintained at 27 ± 2 °C and 80 ± 2% relative humidity. Cotton pads soaked in 5% glucose and 5% multivitamin syrup were provided immediately after blood-feeding and changed daily until the mosquitoes were dissected to assess malarial infection. A detailed protocol is shown in the supplementary information (Additional file 1: File S1).

In additional experiments, patient plasma was separated from *P. vivax*-infected red blood cells to assess the effect of host plasma on mosquito infection. The blood samples were divided into two equal volumes of 3 mL in two tubes. One aliquot was used as whole blood (unwashed) and the second aliquot was processed for serum replacement (washed). Washed blood samples were prepared by centrifuging *P. vivax*-infected patient blood at 500 × *g* for 5 min at 37 °C, washing twice with two volumes of serum-free RPMI1640 medium (Gibco), and replacing the serum with an equal volume of heat-inactivated human naïve AB serum (Sigma-Aldrich, #H4522). Blood samples were kept at 37 °C during the processes until feeding experiments.

### Mosquito dissection and parasite counting

For each feeding experiment, 5 to 20 mosquitoes were dissected 7 and 14 days after blood feeding to assess oocyst and sporozoite infections in the midgut and salivary glands, respectively. Each mosquito midgut was stained with 2% mercurochrome, and the number of oocysts per midgut was counted under a light microscope at 400 × magnification. If the first five midguts were negative for oocysts, 15 more mosquitoes were dissected to confirm oocyst infection. The mean oocyst number for each experiment was typically calculated from five dissected mosquitoes, although some were from 20 mosquitoes. The salivary gland sporozoites were examined under a phase-contrast microscope (Carl Zeiss Axio Lab. A1) at 400 × magnification. For each sporozoite-positive mosquito batch, salivary glands from five mosquitoes were dissected, pooled into microcentrifuge tube containing 50 μL sterile serum-free RPMI1640 medium (KD medical, USA, #CUS-0645), and ground with a sterile pestle. The released sporozoites were then counted using a Neubauer chamber haematocytometer to calculate the average number of sporozoites per mosquito.

### Mosquito survival rate

The mosquito survival rate was calculated by comparing the number of viable mosquitoes 7 and 14 days post-blood feeding by the number of engorged mosquitoes on day 0.

### Statistical analysis

Results were analysed using GraphPad Prism 7.0e software. The percentage blood feeding and mosquito survival rate post-feeding were calculated, and group differences were analysed using paired t-tests. The mosquito infection rate was determined as the proportion of oocyst/sporozoite-positive mosquitoes to the total number of mosquitoes dissected. The infection intensity was determined as the number of oocysts/sporozoites in individual mosquitoes. The Wilcoxon matched-pairs signed-rank test was used to assess differences in oocyst infection rates, average oocyst loads, sporozoite infection rates, and average sporozoite loads between assays. Statistical significance was accepted when the *P*-value was < 0.05. Spearman’s correlation coefficient was used to evaluate the relationship between gametocytaemia and sporozoite production. Correlations between oocyst and sporozoite infection rates and between average oocyst and sporozoite loads were also tested using Spearman’s correlation.

## Results

The MESA-ICEMR team at NIMR-Goa has initiated techniques to conduct infection studies using *An. stephensi* [12] but the procedures needed standardization to set up a continuous reliable supply of *P. vivax* sporozoites. Some initial feeding experiments failed to produce infected mosquitoes, as indicated by the absence of midgut oocysts. Mosquito feeding procedures were therefore re-examined.

**Fig. 1:**
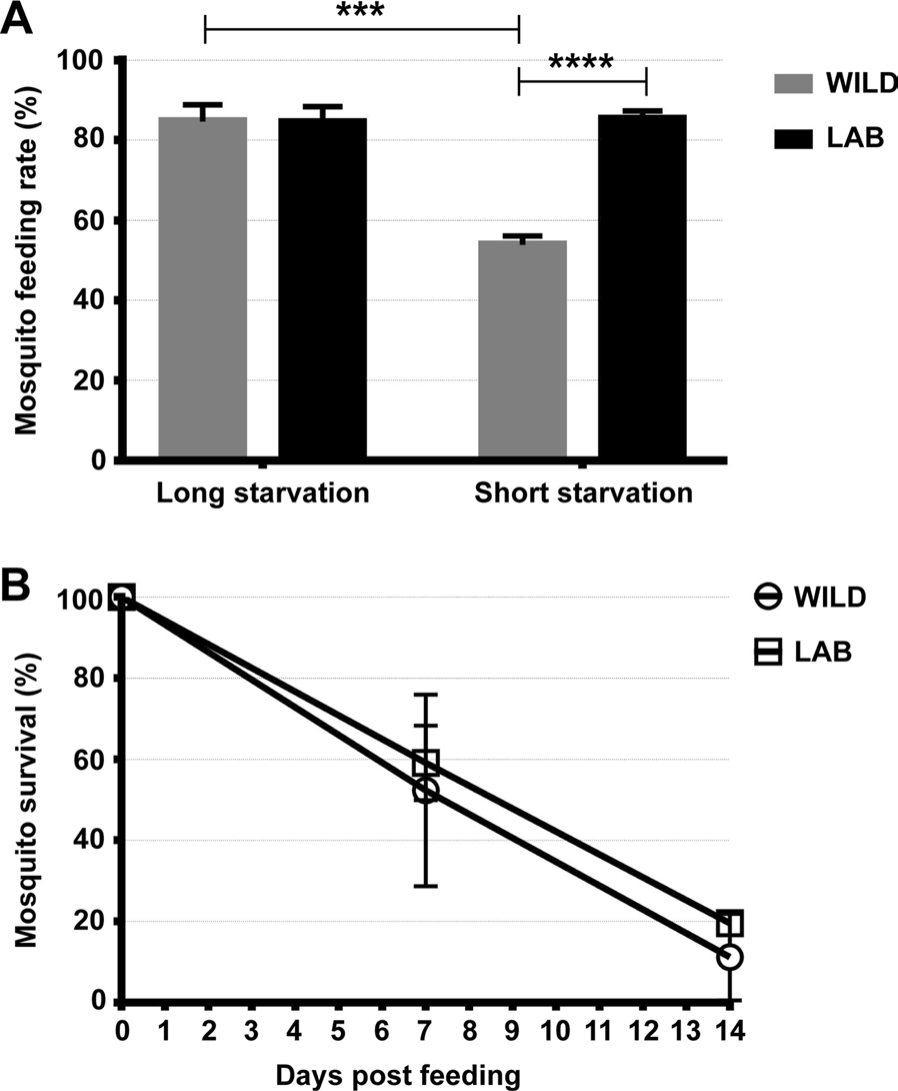
Mosquito-feeding rates and survival rates of wild and laboratory-colonized *Anopheles stephensi.* Blood-feeding rate was determined by the proportion of engorged mosquitoes relative to total mosquitoes tested. **A** Effect of mosquito starvation period on feeding rates of wild and laboratory-colonized mosquitoes. Long starvation is 16–21 h before feeding. Short starvation is 4–6 h before feeding. Data are represented as mean ± standard deviations (SDs) from three independent experiments. Means were compared using *t*-test: ***P < 0.001; ****P < 0.0001. **B** Survival rate of mosquitoes after malaria infection was determined by comparing the number of viable mosquitoes 7 days and 14 days post-patient blood feeding to the total number of engorged mosquitoes on day 0. Paired-infections with the same *P. vivax* blood donor were performed with laboratory-colonized mosquitoes compared to age-matched wild mosquitoes (N = 3). Data are shown as means and SDs

Duration of mosquito starvation was tested as an important variable for good feeding rates. One group of mosquitoes was starved for a short period (4–6 h), and another group was starved longer (16–21 h). Feeding rates, determined by comparing the number of blood-fed mosquitoes to the total number of mosquitoes tested under each condition, were recorded and further compared between wild and laboratory-colonized mosquitoes. After long starvation, wild and laboratory-colonized mosquitoes showed a similar rate of blood feeding at 84.7 ± 4.2 and 84.5 ± 3.9%, respectively (Fig. 1A). After short starvation, the blood-feeding rate of wild mosquitoes dropped significantly to 53.8 ± 2.3% compared with those subjected to the long starvation (*P* = 0.0004), and the rate was significantly different from that of the laboratory-colonized mosquitoes (85.3 ± 2.0%, *P* < 0.0001) (Fig. 1A). The laboratory-colonized mosquitoes showed a similar feeding rate after both starvation times, which suggests that they had previously adapted to the glass membrane feeders during laboratory-adaptation; they achieved and maintained a better feeding rate after the shorter starvation period than the wild mosquitoes.

After malaria infection, high mortality rates were observed in both the wild and laboratory-colony mosquitoes. On day 14 post-infection, only 11.2 ± 10.7% of wild and 19.6 ± 2.3% of laboratory-colonized mosquitoes were available for sporozoite dissection (Fig. 1B). Similarly, there was a dramatic loss of uninfected adults in both the laboratory colony and wild mosquitoes during the experiments. Importantly, hepatocyte cell cultures [15] showed contamination shortly after they were inoculated with sporozoites from the surviving mosquitoes. These results suggest that the mosquitoes were unhealthy and carried microbes that contaminated the cultures.

**Fig. 2:**
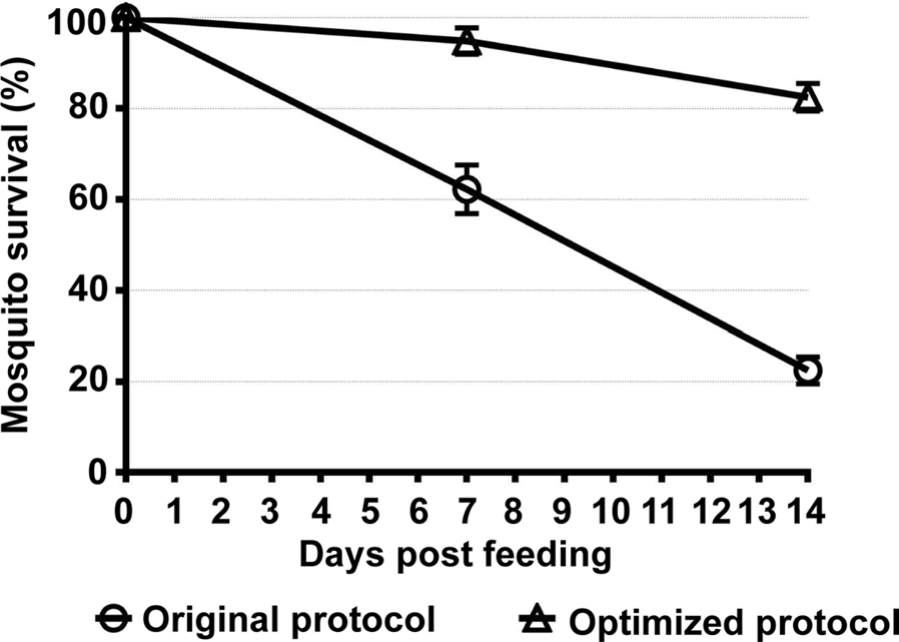
Mosquito survival rate of laboratory-colonized *Anopheles stephensi*. Three batches of laboratory-colonized mosquitoes rearing by original protocol (N = 3) and four batches from optimized protocol (N = 4) were observed and each mosquito batch was independently fed using different *P. vivax* isolates. Data are shown as means and SDs

The operating procedures for insectary rearing and maintenance were, therefore, optimized to ensure the highest possible mosquito quality and longevity. Two groups of mosquitoes were reared under two protocols, original and optimized, and then allowed to feed on *P. vivax* to examine the effects of different mosquito rearing on their survival. Specifically, three batches of laboratory-colonized mosquitoes (N = 3) were reared using the original protocol and four batches (N = 4) were reared with the optimized protocol. Each mosquito batch was independently fed using different *P. vivax* isolates. As shown in Fig. 2, the optimized rearing protocol significantly increased the longevity of *P. vivax*-infected mosquitoes. Survival rates at day 14 post-feeding rose from 22.5 ± 3.0% to 82.5 ± 3.0% (*P* < 0.0001). Key modifications to the original protocol were in the larval rearing conditions and larval/adult diets. In the original protocol, tap water was used for rearing larvae at a density of 400–450 larvae per rearing tray. In the optimized protocol, reverse osmosis water was used to rear 200–250 larvae per tray. Tetramin fish food was utilized in the optimized protocol as the standard food source for larvae instead of the locally made Cerelac power (Nestle) and fish food (1:1) mixture. In addition, in the optimized protocol, 10% glucose solution was diluted to 5% and supplemented with 5% multivitamin syrup to maintain the adults.

**Fig. 3:**
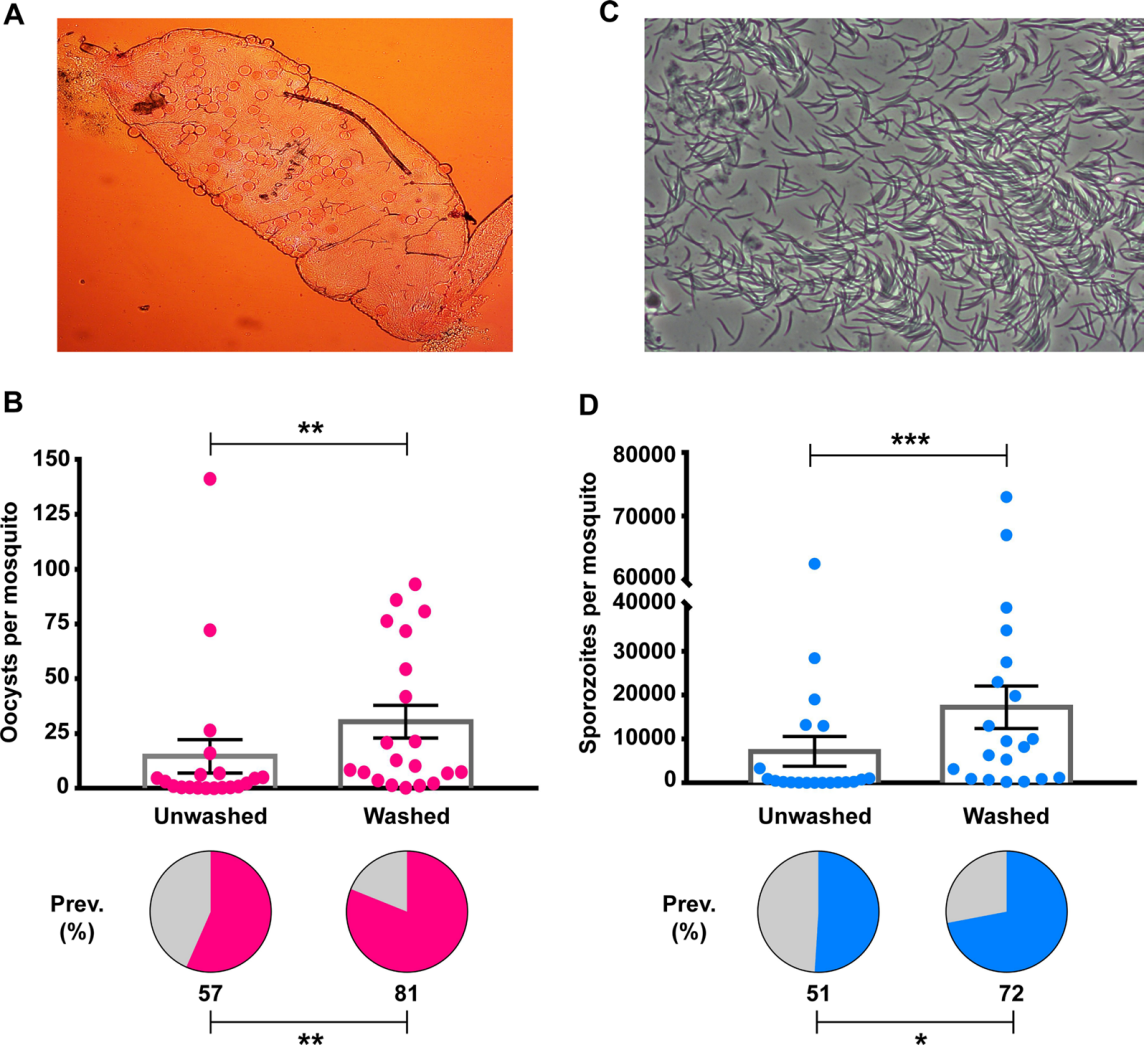
Effect of serum replacement on mosquito infections. Serum replacement was prepared by replacing patient plasma with naïve serum. Mosquitoes were separated into two groups; one group was fed on whole blood and the other, with serum-replaced blood. **A** Mercurochrome stained midgut oocysts, dissected 7 days post feeding. **B** Effect of serum replacement on oocyst infection prevalence and intensity. **C** Salivary gland sporozoites dissected 14 days post feeding. **D** Effect of serum replacement on sporozoite infection prevalence and intensity. Each circle represents a mean number of oocysts/sporozoites in individual mosquito. Pie charts represent the prevalence of infections. Wilcoxon matched-pairs signed-rank test was used to assess the differences in infection prevalence and intensity; *P < 0.05; **P < 0.01; ***P < 0.001

Under the MESA-ICEMR programme, mosquito-feeding experiments were conducted at the field insectary in Goa to optimize the study of parasite-vector interactions. Whole blood from malaria patients was used for mosquito feeding. There was a concern that patient plasma in an endemic setting may have transmission-blocking factors that interfere with the infectivity of gametocytes in mosquitoes. For this reason, the effect of serum replacement on *P. vivax* infections was examined using high-quality mosquitoes obtained with the optimized rearing protocol. To achieve this, 25 independent feeding experiments with different *P. vivax* patient isolates were conducted. Mosquitoes fed on whole blood (unwashed) and serum-replaced (washed) blood at similar rates of 85.3 ± 2.0% and 89.8 ± 5.5%, respectively (Additional file 2: Fig. S1). Five of the 25 experiments (20%) failed to produce mosquito infections from both unwashed and washed blood conditions, and all five negative cases were excluded from further analysis. Among the negative cases, one had a mixed *P. vivax*/*Plasmodium faciparum* infection, as confirmed by a rapid diagnostic test (RDT) at the insectary. Table 1 presents oocyst infection prevalence (batches with one or more oocysts in the mosquito midgut), sporozoite infection prevalence, and mean values of oocyst and sporozoite density in the 20 batches with positive infections. Figure 3A and 3C show representative images of *P. vivax* midgut oocysts and salivary gland sporozoites, respectively. A wide range of oocyst infection rates was observed in both the unwashed (range of 0–100%) and washed (range of 20–100%) blood-feeding groups. The mean oocyst density per mosquito varied from 0 to 141.2 and 0.2 to 93.2 for unwashed and washed blood, respectively. However, the level of individual infections were different in mosquitoes fed on washed *versus* unwashed blood. For unwashed blood, 56.5 ± 33.6% of the mosquitoes were infected with a mean value of 14.6 ± 7.61 oocysts per mosquito (Table 1 and Fig. 3B). Mosquitoes fed on serum-replaced samples had significantly higher oocyst infection rates (81.2 ± 22.3%, *P* = 0.0022) and higher mean oocysts per mosquito (30.4 ± 7.47, *P* = 0.0065) than those fed on unwashed blood (Table 1; Fig. 3B). The sporozoite infection rate was significantly higher in the washed blood group (72.0 ± 27.1%) than in the unwashed blood group (51.0 ± 39.3%, *P* = 0.0476). Similarly, the mean number of sporozoites per mosquito was significantly higher in those fed serum-replaced blood (17,225 ± 4831) compared with those given whole blood (7170 ± 3404) (*P* = 0.0002) (Table 1; Fig. 3D).

**Table 1:** Laboratory-colonized *An. stephensi* infection with Indian *P. vivax* infected blood

		% Gametocytemia	Oocyst infection		Sporozoite infection	
			% Infected mosquitoes	Oocysts per mosquito	% Infected mosquitoes	Sporozoites per mosquito
Unwashed blood	N = 20	0.20 ± 0.12 (0.059–0.58)	56.5 ± 33.6 (0–100)	14.6 ± 7.61 (0–141.2)	51.0 ± 39.3 (0–100)	7170 ± 3404 (0–62,500)
	*N = 12	0.17 ± 0.08 (0.06–0.33)	72.5 ± 27.3 (20–100)	23.7 ± 12.2 (0.4–141.2)	79.2 ± 19.3 (60–100)	11,875 ± 5322 (50–62,500)
Washed blood	N = 20	0.19 ± 0.13 (0.04–0.58)	81.2 ± 22.3 (20–100)	30.4 ± 7.47 (0.2–93.2)	72.0 ± 27.1 (20–100)	17,225 ± 4831 (200–73,000)
	*N = 15	0.17 ± 0.10 (0.04–0.48)	89.5 ± 13.4 (60–100)	39.6 ± 8.77 (1.4–93.2)	85.3 ± 14.1 (60–100)	22,562 ± 5837 (275–73,000)

% Gametocytemia and % Infected mosquitoes are expressed as mean ± SD and range

% Gametocytemia of washed blood samples were determined after blood washing process

Oocysts per mosquito and sporozoites per mosquito are expressed as mean ± SEM and range

^*^Values for batches with ≥ 50% sporozoite-infected mosquitoes

**Fig. 4:**
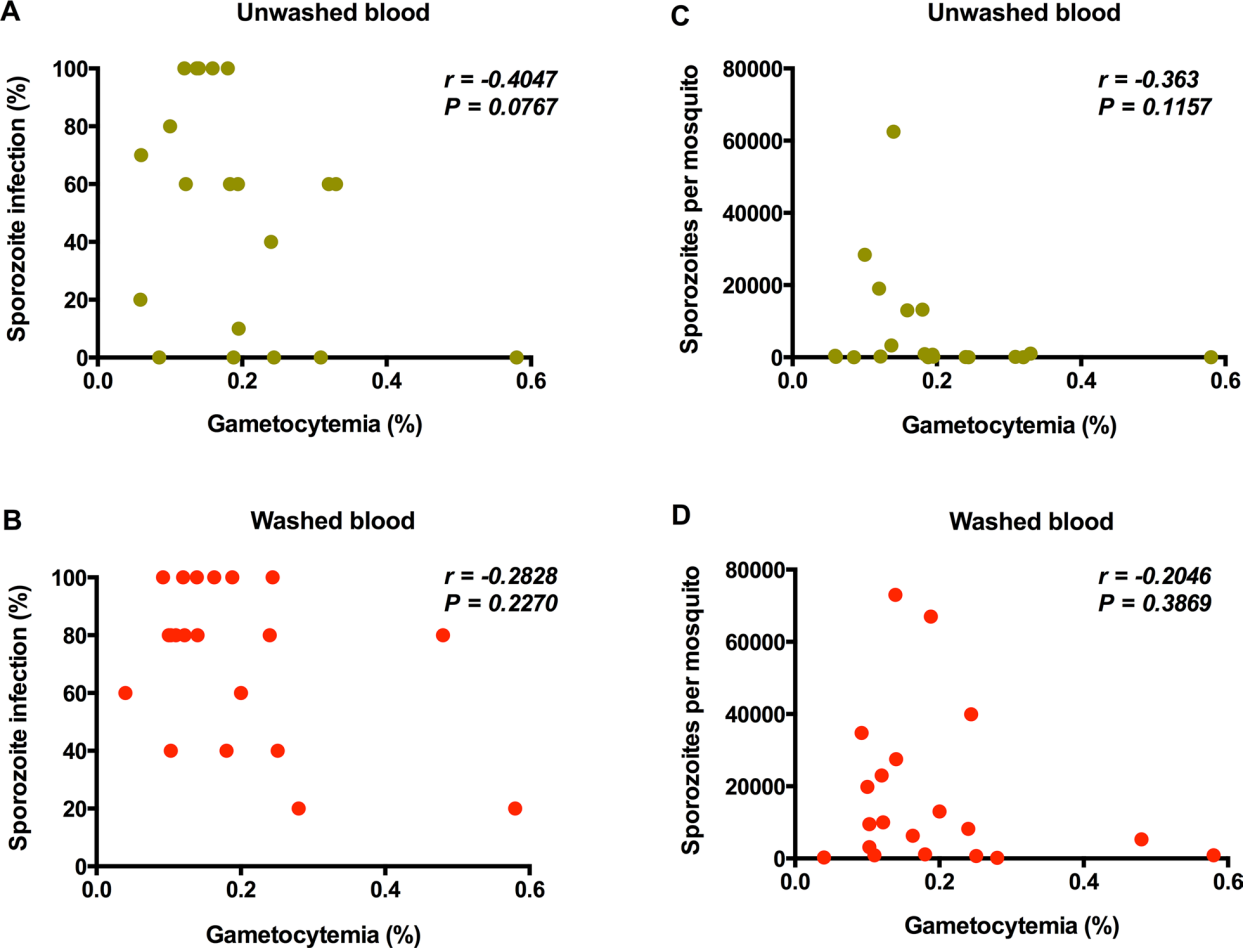
Correlation between gametocytaemia and sporozoite production. Correlation between gametocytaemia and sporozoite infection rate are shown in **A** for unwashed blood and **B** for washed blood feedings. Correlation between gametocytaemia and sporozoite load are shown in **C** for unwashed blood and **D** for washed blood feedings. Spearman’s correlation coefficient was used to evaluate the relationship between the data

**Fig. 5:**
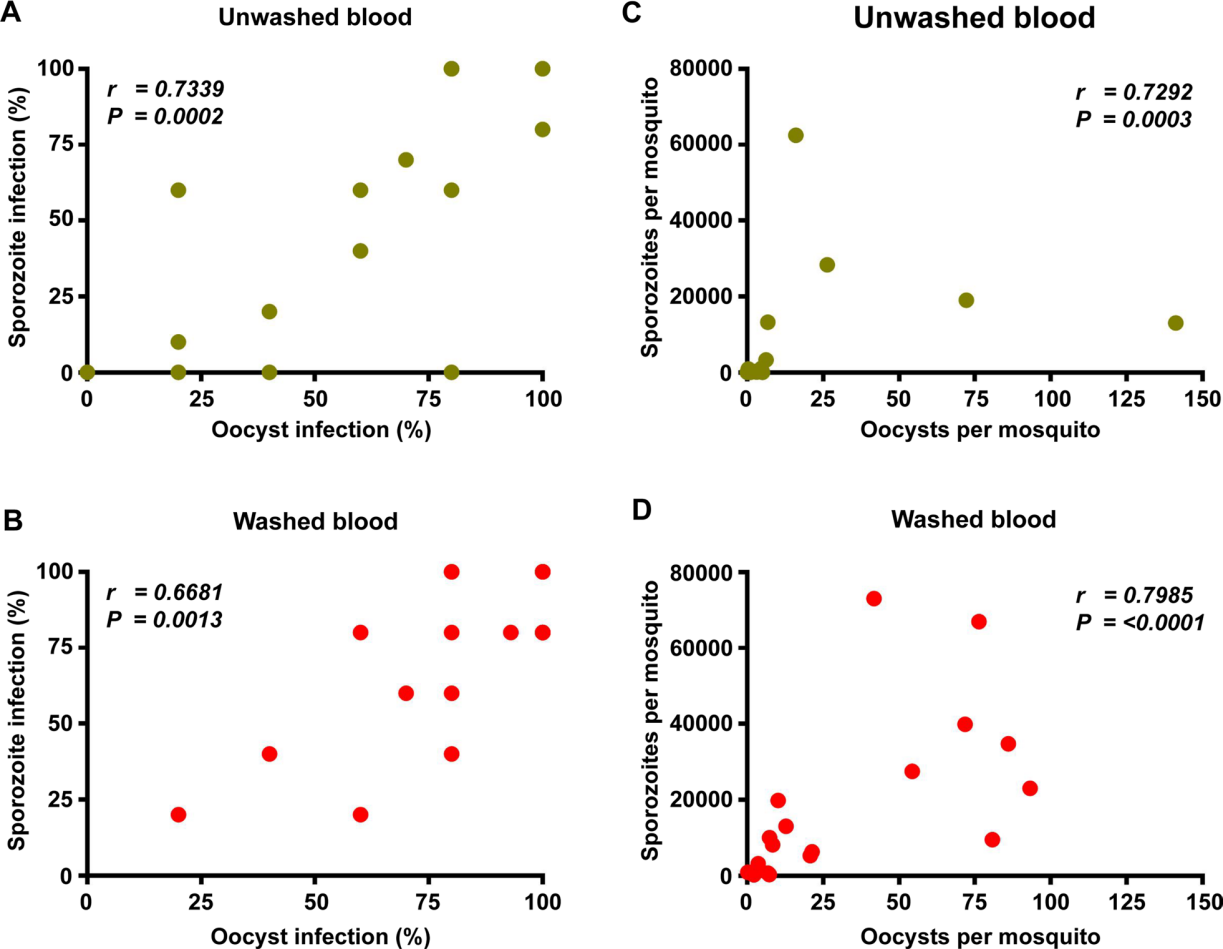
Correlation between oocyst infection and sporozoite production. Mosquito infection rate and intensity for experimental mosquito feedings using unwashed and serum replaced (washed) blood were measured. Correlation between sporozoite infection and oocyst infection rates are shown in **A** for unwashed blood and **B** for washed blood feedings. Correlation between oocyst and sporozoite loads for unwashed and washed blood are shown in (**C**) and (**D**), respectively. Correlations between oocyst and sporozoite infection rates and between average oocyst and sporozoite loads were tested using Spearman’s correlation

The patient blood used in this study showed gametocytaemia ranging from 0.059 to 0.58%. There were no correlations between gametocytaemia and the prevalence of sporozoite infection or density of infection (mean sporozoites per mosquito) in both the unwashed (Fig. 4A, C) and washed blood-fed groups (Fig. 4B, D). However, the percentage of mosquitoes carrying oocysts strongly correlated with the percentage of mosquitoes with developing sporozoites in both the unwashed (*r* = 0.7339, *P* = 0.0002) (Fig. 5A) and washed blood (*r* = 0.6681, *P* = 0.0013) (Fig. 5B) groups. Additionally, a significant correlation was detected between oocyst and sporozoite loads in the corresponding batches of both the unwashed (*r* = 0.7292, *P* = 0.0003) (Fig. 5C) and washed blood-fed mosquitos (*r* = 0.7985, *P* < 0.0001) (Fig. 5D).

**Fig. 6:**
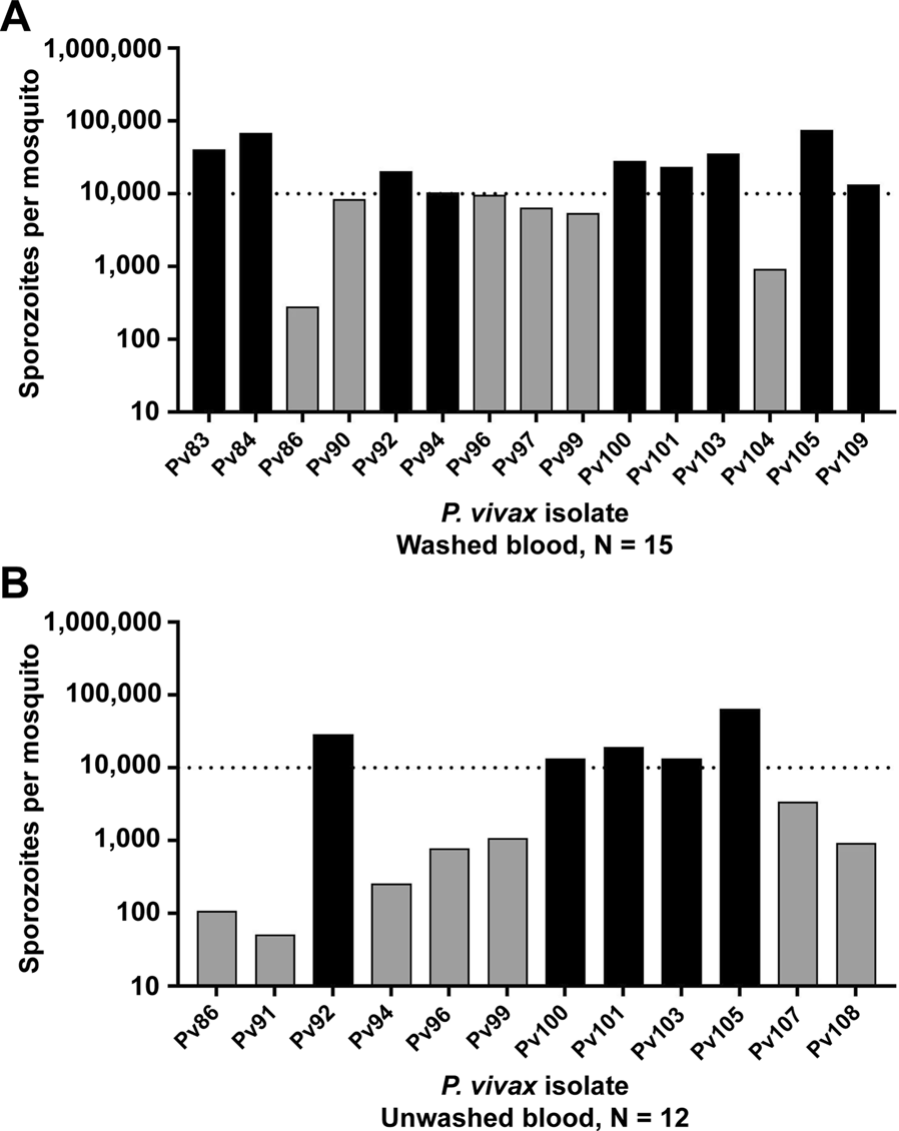
Sporozoite loads of batches with ≥ 50% sporozoite-infected mosquitoes. Mean sporozoites per mosquito values for mosquito feeding batches using serum replaced (washed) and unwashed blood are shown in (**A**) and (**B**), respectively. Black bars represent batches with ≥ 10,000 sporozoites per mosquito

The number of sporozoites in individual infected mosquitoes varied with different batches of patient samples. During sporozoite production, only the mosquito batches with the highest sporozoite loads are useful for further liver-stage studies. High loads reduce the proportion of mosquito debris and microbial contamination during salivary gland dissection. To increase the amount of sporozoites for the liver-stage assay, mosquito batches that were ≥ 50% sporozoite positive were chosen for analysis. Twelve batches (60%, 12/20) fed with unwashed blood, and 15 (75%, 15/20) with washed blood, exhibited this level of sporozoite infection (Table 1). In the washed-blood group, 9 of the 15 batches (60%) yielded ≥ 10,000 sporozoites per mosquito (Fig. 6A). In the unwashed-blood group, 5 of the 12 (42%) mosquito batches reached this sporozoite rate (Fig. 6B). Using this selection criterion, the average number of sporozoites per batch increased from ~ 7100 to 11,875 ± 5322 (range 50–62,500) in the unwashed experiments and from ~ 17,000 to 22,562 ± 5837 (range 275–73,000) in the washed experiments (Table 1). Overall, out of four batches of mosquito infections recruited for the experiments, 3 batches from the washed and 2.4 batches from the unwashed group passed the selection criterion, with ≥ 50% sporozoite infection. Of the selected batches, there was a probability of having at least two cases from the washed samples providing ≥ 10,000 sporozoites per mosquito, while only one case from the unwashed samples would be expected to have this sporozoite level. In other words, the success rates for producing high-sporozoite loads using washed and unwashed blood-feedings were 50% (2/4 batches) and 25% (1/4 batches), respectively.

## Discussion

In recent years, there has been increasing interest in the liver stages of *P. vivax* in India [16, 17]. Research is underway at a field site in Goa to produce *P. vivax* sporozoites from clinical isolates [11, 12]. These will be used in future for liver-stage studies to better understand the *P. vivax* population in India. The present report focuses on improving procedures for generating high-quality sporozoite-infected mosquitoes.

Success of mass production of *P. vivax* sporozoites (e.g., ≥ 50,000 per mosquito) through artificial membrane-feeding techniques [5, 7] is strongly dependent upon the robust rearing of good quality *Anopheles* mosquitoes. In the NIMR Goa/MESA ICEMR laboratory, a few thousand *An. stephensi* mosquitoes are routinely produced every week for research on malarial infections, and over 38 mosquito batches were provided for the *P. vivax*-feeding experiments described in the present study. In the initial phase, mortality rates of mosquitoes, both with and without *P. vivax* infection, were extremely high and very few mosquitoes were available on the day of sporozoite dissection. This setback highlighted the poor quality and health of the mosquitoes produced in the insectary. A critical part of the mosquito-rearing process is the maintenance of the larvae [18]. Therefore, the larvae-rearing procedures were examined to identify ways to increase mosquito fitness, especially after malaria infection. *Anopheles stephensi* larvae prefer clean water habitats and chlorine-free water with low nitrite and high phosphate levels [19]. Chlorine-free water was easily prepared by standing tap water for a few days before use to allow the chlorine to dissipate. Nevertheless, many other excessive chemicals in tap water, such as copper, nitrates, nitrites, and silicates, could be detrimental to larvae. The mortality rate of larvae reared in tap water has been reported to be as high as 90% [20]. To avoid detrimental outcomes, local tap water was replaced with reverse osmosis water in the system. When larvae were reared in reverse osmosis water and the numbers limited to between 200 and 250 larvae per rearing tray, the survival rate of infected mosquitoes increased from 23 to 51% on day 14 post-infection. The larval density could not be excessively high, as it is known to substantially impact larval survival [18]. In the laboratory, mosquito larvae raised in high-density conditions face greater competition for food and space, which in turn, reduces larval survival and affects adult development [21–23]. In addition, a high density of larvae may expose them to waste toxins and water contamination from overfeeding, and frequent physical contact between larvae can also induce stress [24]. When adjusted to 200–250 larvae per tray, the density was around 0.3 larvae/sq cm, which is similar to the optimum rearing density reported elsewhere [18]. These results confirm that environmental conditions are critical for the successful rearing of *An. stephensi* mosquito larvae.

The quality of the larval diet is another important factor that can affect development and adult longevity [25]. The Cerelac fish food mixture from India has been used for decades at the insectary for the routine rearing of mosquito colonies. However, when Tetramin fish food (a standard insectary larval diet) was provided during larval development, the survival rate of the adult mosquitoes was considerably higher. This result concurs with a study on *Anopheles gambiae* that showed that Tetramin fish food performed better than the Cerelac fish food mixture for rearing mosquito larvae [26]. For adult female mosquitoes, plant sugars are an essential energy source, affecting their longevity, fecundity, host-seeking behaviour, and ability to transmit disease [27]. Commercially available processed plant sugars, glucose and sucrose, are frequently used to rear mosquitoes in the laboratory [18, 28]. Combining sugar with dietary supplements, such as antioxidants [29, 30], amino acids [31], and corn pollen [32], which contains amino acids, minerals and vitamins increases mosquito longevity. Children’s multivitamin syrup used in this study contains important vitamins for larval mosquitoes: thiamine (vitamin B1), riboflavin (vitamin B2), pantothenic acid (vitamin B5), and nicotinamide [33, 34]. Although little is known about the effects of vitamins on adult mosquitoes, multivitamin syrups are routinely added to sugar diets for adult anophelines in some insectaries [35–38]. The addition of multivitamin syrup to a sugar diet also increased the longevity and health of male mosquitoes [38]. Indeed, the combination of multivitamin syrup and sugar used in this study led to the highest mosquito survival rates.

The effects of anticoagulants on *P. vivax* oocyst development in South American *Anopheles* mosquitoes have been reported previously [9, 39]. Heparin is recommended as the anticoagulant of choice for the blood used in artificial membrane-feeding assays, because it is associated with the highest oocyst and sporozoite yields [9, 39]. However, acid citrate dextrose was employed, as the anticoagulant in the present work, as resources were shared with other studies in which heparin had to be avoided. The effect of heparin on *P. vivax* development in laboratory-colonized *An. stephensi* will be further investigated in the future: there are reasons to believe that sporozoite productivity can be further improved.

Laboratory-colonized mosquitoes are widely used in mosquito-feeding assays and for the production of *P. vivax* sporozoites [7–9, 12, 40, 41]. This study showed that the colony mosquitoes adapted to glass membrane feeders achieved a better feeding rate in the laboratory, even after a shorter starvation period. The short starvation time requirement of the laboratory-colonized mosquitoes increases the robustness of the membrane-feeding experiments. At GMC hospital, patients are generally enrolled during normal working hours with blood collection usually occurring in the afternoon. To coordinate two activities, mosquitoes could be starved each morning, not long before blood feeding. A shorter starvation period of 3 h has been reported before the membrane feeding of *An. stephensi* with *Plasmodium* parasite blood [42]. The higher feeding efficiency of the colony compared with wild mosquitoes has also been reported for *Anopheles arabiensis*, and a longer starvation period is also preferred for wild-caught mosquitoes [43]. This finding guides the use of laboratory-colonized mosquitoes in feeding experiments, which offers a significant advantage over the use of wild mosquitoes in terms of logistics, ease of maintenance and handling, and scaling-up flexibility and reproducibility of the assays [12].

This study demonstrated the feasibility of scaling up *P. vivax* sporozoite production in *An. stephensi* at field sites in South West India. Although the number of sporozoites obtained varies with different batches of patient blood samples, it could be as high as 73,000 sporozoites per mosquito. Several factors are known to influence the variation in mosquito infection, including gametocytes density [44] and the presence of host immunity [45]. A positive association between gametocyte density and infectivity has been discovered in several other studies [9, 43, 46] but was not observed in the present study. This finding may reflect the presence of transmission-blocking components in the blood of Indian patients. GMC is a major hospital in South West India; almost all malaria patients visiting or admitted to the hospital are migrant workers from different parts of the country with diverse genetic backgrounds and different histories of prior exposure to malaria [13]. This diversity in the patient population and the data from recent work showing high malarial immunity in patients at GMC [47] may explain the wide range of sporozoite infection rates, especially from whole-blood-feeding experiments. The increase in mosquito infection and sporozoite levels seen with serum replacement underscores the importance of patient plasma on mosquito-feeding experiments [9, 46]. Therefore, serum replacement of patient blood before mosquito feeding is necessary for the consistent and high-level production of sporozoites, especially in endemic areas where there is a high diversity of human host factors. About 20% of the patient blood enrolled in this study completely failed to produce mosquito infections in both whole-blood and serum replacement experiments. Interestingly, these patients had taken antipyretics, e.g., paracetamol, to reduce fever approximately 4–12 h prior to blood collection. There are no reports on the anti-parasitic effects of antipyretics used to treat fever in malaria patients. Therefore, in future, it will be of interest to assess the potential transmission-blocking effects of antipyretics in controlled experiments. In addition, the strong correlation between oocyst load and sporozoite production after whole-blood feeding indicates that host factors have less influence on the development of *P. vivax* in mosquitoes than the transmission stage of the parasites. Taken together, these data point to specific variables in human host serum contents that influence parasite development in the vector.

In this study, mosquito batches with ≥ 50% sporozoite infection rates were considered optimal for sporozoite production. Using this selection criterion, serum replacement not only increased the percentage of sporozoite-carrying mosquitoes from ~ 72 to ~ 85% but also yielded a higher number of sporozoites per mosquito (22,562; range 275–73,000). This sporozoite rate was comparable to the levels that have been reported from laboratories using *An. dirus* [7] and *An. cracens* [8]. Of the sporozoite-positive batches, 60% resulted in the production of ≥ 10,000 sporozoites per mosquito. Large numbers of sporozoites are generally required for liver-stage infection, i.e., ~ 250,000 sporozoites per well in 8-well format or ~ 25,000 per well in 96-well format. Therefore, operationally, only mosquitoes with ≥ 10,000 sporozoites were chosen for liver-stage experiments. This was to avoid excessive mosquito debris that increases the risk of contamination in liver cell cultures. With these cautionary practices, in a few liver-infection trials, no contamination was detected even when salivary gland sporozoites from up to 20 mosquitoes were added to a well of an 8-well chambered slide. This was indicative of the high quality of the sporozoite-infected mosquitoes obtained, and this reduced the need for antibiotics in insectary processes.

This is the first report on the optimization of *P. vivax* sporozoite production in *An. stephensi* at a field site in India. A number of strategies emerged to continuously generate uniform and fit mosquitoes for *P. vivax* sporozoites production. First, laboratory-colonized mosquitoes were chosen after they had adapted to the membrane-feeding procedure and thus were readily available for experimental infections, compared to wild mosquitoes. Second, the modified rearing protocol improved mosquito survivorship and increased the number of sporozoite-infected mosquitoes available for liver-infection experiments. Finally, serum replacement of donor blood also improved mosquito infections and sporozoite loads. In future, it will be important to find additional treatments and conditions to further improve the effectiveness of the sporozoites produced for liver infection. A recent report has shown low liver-infectivity of Indian *P. vivax* sporozoites in the currently available liver platforms [17]. *Plasmodium vivax* infection in the classical host HC-04 cells was also observed in the present study, but at a very low frequency. This clearly indicates that there is room for improvement in such studies. For example, batches of primary human hepatocytes can be pre-selected for their susceptibility to Indian *P. vivax* sporozoite infection [48]. In addition, utilizing a new hepatocyte cell line for *P. vivax* liver-stage assay [49] may also offer opportunities to experimentally characterize hypnozoites in the Indian population. The inability to reliably and fully develop Indian *P. vivax* isolates in cultured liver cells requires more effort but it is also important to remember that attempts to propagate blood-stage forms of *P. vivax* have also been largely unsuccessful. There may be general solutions to *P. vivax* propagation that will be needed for all *P. vivax* stages. Moreover, the protocols established here not only detail the steps taken to establish *P. vivax* sporozoite production to initiate a liver-stage programme but they can also lead to a valuable resource for other future researchers who require a complete sporogonic cycle of the parasites for studies on mosquito immunology and/or transmission blocking function and vaccines.

## Conclusion

Optimized mosquito-rearing and mosquito-feeding protocols were established at a field setting in South West India. The procedures consistently produced high-quality *An. stephensi* with salivary glands infected with high *P. vivax* sporozoite loads. Thus, these standardized procedures for the robust production of *P. vivax* sporozoites can benefit studies of *P. vivax* liver stage infections in Indian populations. These efforts will help to advance research on hypnozoite biology and the development of more effective anti-relapse interventions.

## Supplementary Information

**Additional file 1**: **File S1**. Details of mosquito rearing and mosquito infection by membrane feeding.

**Additional file 2**: **Fig. S1**. Effect of serum replacement on mosquito feeding rate of laboratory-colonized An. stephensi.

## Data Availability

All data generated during this study are included in this published article and the additional files. Data are available from the corresponding author on reasonable request.

## Abbreviations

DMID: Division of Microbiology and Infectious Disease
GOI HMSC: Government of India Health Ministry Screening Committee
GMC: Goa Medical College and Hospital
ICEMR: International Center of Excellence for Malaria Research
km: Kilometer
MESA: Malaria Evolution in South Asia
NIAID: National Institute of Allergy and Infectious Diseases
NIH: US National Institutes of Health
NIMR: National Institute of Malaria Research
RDT: Rapid Detection Test
UW: University of Washington
