# Supplementary material for: Optimization of Plasmodium vivax sporozoite production from Anopheles stephensi in South West India
Source: Malar J. 2021 May 18;20:221. doi: 10.1186/s12936-021-03767-2 (PMC8129701; doi:10.1186/s12936-021-03767-2)
Supplement: Supplementary file 1 — Additional file 1: File S1. Details of mosquito rearing and mosquito infection by membrane feeding. [file 12936_2021_3767_MOESM1_ESM.pdf]

Supplementary File S1.

Details of mosquito rearing and mosquito infection by membrane-feeding.

A. Rearing and maintenance of *Anopheles stephensi* mosquitoes

Location: The rearing and maintenance of a continually cycling colony of *An. stephensi* mosquitoes was carried out at the US NIH MESA ICMR facilities built within the National Institute of Malaria Research (ICMR), Goa, India.

Laboratory conditions: A mosquito culture room simulated natural climatic conditions by maintaining a temperature of 27 ± 2°C, relative humidity of 70 ± 5% and a photoperiod of 12 h of day and night cycle. Temperature and humidity of the lab were monitored on a daily basis and recorded three times a day, once in the morning, once in the afternoon, and finally in the evening.

Raising larvae: The larvae were reared in plastic trays (25 x 30 x 4 cm) with 1.5 L reverse osmosis (RO) water at a density of 200-250 larvae per tray. Approximately 10 mg of powdered TetraMin Tropical Flakes was given to the growing larvae, once a day, until the development of pupal stage (approximately 6-8 days). On every alternate day, the water from the rearing trays was replaced with fresh RO water. The dead larvae were also counted and removed for each rearing tray. The health of the larvae in each tray was monitored on a daily basis. If the larval mortality in a tray was > 5%, we did not proceed with further rearing in that particular tray and discarded its contents.

Transformation to pupae and adult mosquitoes: Each day, pupae were counted and collected in small 500-mL capacity plastic bowls containing 200 mL of RO water. They were kept inside a closed net cage until emergence of adults was seen (approximately 2 days).

Maintaining adult mosquitoes: Upon adult emergence, each cage was provided with cotton pads soaked with 5% glucose and 5% multivitamin syrup solution, and these pads were replaced daily.

Blood feed setup: Four days post emergence, 100-200 female mosquitoes were carefully aspirated and transferred into a plastic feeding cup covered with mesh netting, and secured by a rubber band. The mosquitoes were starved inside the

Percival chambers at 27 +/-2°C and 80 +/- 2% relative humidity for 3-4 h prior to blood feeding. Glass feeders with a capacity of 3 mL, connected to a circulating warm water bath, were used for feeding mosquitoes. For this, water at 37°C was circulated between the inner blood-containing chamber and the outer chamber of the glass feeder. Rubber tubing connected the glass feeders to the two side nozzles of the circulating warm water bath. A small piece of parafilm was stretched and wrapped around the wider end of the glass feeder as a membrane that mimicked warm human skin. The glass feeder was placed on top of the meshed netting covering the mosquito cup. The feeder was secured in place with electrical insulating tape.

Blood meal: About 2 mL of QNS (quantity not sufficient) blood received from the rotary blood bank, Regional blood transfusion Centre, New Delhi was pipetted into the neck of the glass feeder. The blood went through the feeder neck down to the reservoir until it touched the stretched parafilm membrane. Mosquitoes in the feeder cup, flying under the blood feeder, were allowed to feed, through the parafilm membrane on warm blood for 60 min. The feeding room was maintained at 27 ± 2 °C and 75 ± 2% relative humidity using the water heater throughout the entire feeding period.

Eggs from fed mosquitoes: After 60 min, the fully engorged mosquitoes were separated from the unfed ones and transferred using an aspirator into a new cage for oviposition. A small plastic bowl lined with a 3″ wide strip of filter paper with RO water was placed inside the mosquito cage for 48 h. The eggs, deposited on the moist filter paper, were collected and counted.

Larvae from eggs: About 200 eggs were transferred to the hatching plastic bowls with the help of a fine brush, without damaging the eggs. Further, larvae were reared in the plastic tray until the pupae formed. In this fashion, a continuous cyclic colony of the *An. stephensi* is maintained in the insectary.

B. Mosquitoes feeding with *P. vivax*-infected patient blood

Preparing mosquitoes for parasites: Five to six-day-old female mosquitoes were aspirated and put in plastic cups covered with mesh netting and secured by a rubber band. For each condition, a plastic cup containing 100 female mosquitoes per cup was used for feeding.

Initial mosquito starvation before blood feeds: Mosquitoes were starved inside the Percival chambers maintained at 27 ± 2°C and 80 ± 2% relative humidity.

Duration of starvation was 4-6 h for lab strain of mosquitoes and overnight for wild mosquito populations.

Apparatus for feeding infected blood: Glass feeders with 1.5 mL capacity were prepared for mosquito infections. For this, water at 37°C was circulated between inner blood-containing chamber, and outer chamber of the glass feeder, with the help of rubber tubing fixed to the two side nozzles of a circulating-water bath. A small piece of parafilm was stretched and wrapped around the wider end of the glass feeder (as a membrane mimicking human skin) that would later contain parasite-infected blood. The glass feeder was placed on top of the mosquito cup and secured with a tape.

Parasite-infected human blood: *P. vivax*-infected blood samples were collected in an acid citrate dextrose vacutainer at Goa Medical College and Hospital (GMC). *Plasmodium* infection was confirmed by microscopic analysis of Giemsa-stained thin blood smears. After collecting, the samples were placed in a 37°C thermos flask to prevent gametocyte exflagellation and immediately transported from GMC to the NIMR, Goa insectary. To confirm mono *P. vivax* infection of blood samples (free of *P. falciparum*), an additional rapid diagnostic test (RDT) was performed at the insectary prior to mosquito feeding.

Parasitized blood-meal: One mL of *P. vivax*-infected blood was pipetted into the neck of the feeder. The blood should go all the way through the neck down to the reservoir touching the membrane. In this study, 3 mL of infected blood was used to feed 100 female mosquitoes. Therefore, it required three 1.5-mL capacity feeders for feeding 100 mosquitoes.

Special meals: In some experiments, patient plasma was separated from *P. vivax*-infected blood to assess the effect of host plasma on mosquito infection. The blood samples were prepared by centrifuging *P. vivax*-infected blood at 500 × *g* for 5 min at 37°C, washing twice with two volumes of serum-free RPMI1640 medium (Gibco), and replacing the serum with an equal volume of heat-inactivated human naïve AB serum. Blood samples were kept at 37°C during these processes, until mosquito feeding.

Feeding conditions: Mosquitoes were allowed to feed on *P. vivax*-infected blood for a period of 60 min. The feeding room was maintained at 27 ± 2°C and 75 ± 2% relative humidity. After 60 min, fully engorged mosquitoes were separated from the unfed ones using aspirator. The plastic cups containing fed mosquitoes were placed in the Percival incubators maintained at 27 ± 2°C and 80 ± 2%

relative humidity. Cotton pads containing 5% glucose and 5% multivitamin syrup solution were placed on top of the cups to maintain the infected mosquitoes until dissection. The water bath was turned off and feeders were disconnected from the tubing. The feeders were then soaked in 10% bleach for decontamination.

C. Dissecting of mosquito midguts for oocyst count

Gathering post-feed mosquitoes**:** On day 7 post blood feed, 5-10 mosquitoes from the each cup were aspirated and transferred to individual test tubes. The mouth of the test tube was immediately closed with cotton to avoid the escape of mosquitoes. To anesthetize the mosquitoes, the test tubes containing mosquitoes were placed inside the refrigerator at $0^{o}C$ for at least 5 min until the mosquitoes stop moving. Then, the immobilized/Anesthetized mosquitoes in glass test tubes were transferred to a bucket containing ice cubes.

Dissecting infected mosquito midguts: Using fine-tipped forceps, wings and legs were clipped. Also, the proboscis was cut with a scalpel blade. Each mosquito was placed on a clean glass slide containing a drop of 1X PBS buffer for dissection. Using dissecting needle, the mosquito were held at its thorax, and gently the terminalia was pulled away from the abdomen using a dissecting needle. Care was taken to ensure that the midgut does not detach from the terminal end. Using forceps, malpighian tubes, ovaries, other accessory tissues and debris were removed from the midgut.

Visualizing parasite oocysts in mosquito midguts: The dissected midgut from each mosquito was placed in a small drop of freshly prepared 2% mercurochrome on a new slide and allowed to stain for 1 min. A cover slip was placed over the midguts with gentle pressure to compress the tissue. Excess stain was removed using a tissue paper at the edge of the cover slip. The midguts were examined under a light microscope (Carl Zeiss Axio Lab.A1) at 400x magnification. The number of oocysts in each mosquito midgut was counted and recorded.

D. Dissecting of mosquito salivary glands for sporozoite count

On day 14 post feeding, some mosquitoes were collected in glass test tubes from plastic cups using an aspirator. The mosquitoes were anesthetized by placing them inside the refrigerator at $0^{o}C$ for at least 5 min, or until they stop moving. Then, the immobilized/anesthetized mosquitoes in glass test tubes were

transferred to a bucket containing ice cubes. Using fine-tipped forceps, wings and legs were removed to immobilize the mosquitoes and the proboscis was cut with a scalpel blade. A single mosquito was placed for dissection on a clean glass slide in a drop of 1X PBS buffer. The mosquito was gently held with a needle, and another needle was placed across the neck. The head was then slowly pulled away. The salivary glands were gently detached from the head **(**If the salivary glands remain within the thorax, using a needle, gentle pressure was applied to the thorax towards the mesonatum end to squeeze the glands out and then the salivary gland was detached).

E. Estimating the yield of sporozoites from mosquitoes

For each experiment, the average number of sporozoites per mosquito was typically calculated from five dissected mosquitoes. Salivary glands from five mosquitoes were dissected, pooled into microcentrifuge tube containing 50 μL sterile serum-free RPMI1640 medium (KD medical, USA), and ground with a sterile pestle. The sporozoite suspension was kept on ice. The released sporozoites were then counted using a Neubauer chamber hematocytometer. The hemocytometer was placed on a wet “kimwipe” in a covered petri dish to allow sporozoites to settle for 10 min. If sporozoite load is high, then 1:5, 1:10, or 1:20 dilution was made. The sporozoites were counted in two of the four quadrants of hemocytometer under phase contrast microscope at 400x magnification.

Materials and reagents

Item	Company	Catalog #
TetraMin Tropical Flakes	TETRA GMBH, Melle, Germany	Product # 77101 https://www.tetra-fish.com/products/nutrition/tetramin-tropical-flakes.aspx
Glucose	Heinz India Pvt.Ltd	G37863
Multivitamin syrup solution	Haemo-Vit, Boss Pharmacare Co., LTD., SamutSakhon, Thailand	https://bosspharmacare.com/portfolio/haemovitkids/
Glass membrane feeders	Chemglass Life Sciences LLC, USA	CG-1836, CG-1835
Acid citrate dextrose vacutainer	BD, India	02-684-29
Giemsa-stain	Himedia	S-011
Rapid diagnostic test (RDT)	Zephyr Biomedicals division of Tulip Diagnostics	503150050
RPMI1640 medium (for blood washing)	Gibco	22400089
Human AB serum	Sigma-Aldrich	H4522
PBS buffer, pH 7.4	Gibco	10010023
Mercurochrome solution	Himedia	GRM6029-25G
RPMI1640 medium (for sporozoite collection)	KD Medical, USA	CUS-0645
