# Supplementary material for: Optimization of Plasmodium vivax sporozoite production from Anopheles stephensi in South West India
Source: Malar J. 2021 May 18;20:221. doi: 10.1186/s12936-021-03767-2 (PMC8129701; doi:10.1186/s12936-021-03767-2)
Supplement: Supplementary file 2 — Additional file 2: Fig. S1. Effect of serum replacement on mosquito feeding rate of laboratory-colonized An. stephensi. [file 12936_2021_3767_MOESM2_ESM.pdf]

Supplementary Figure S1

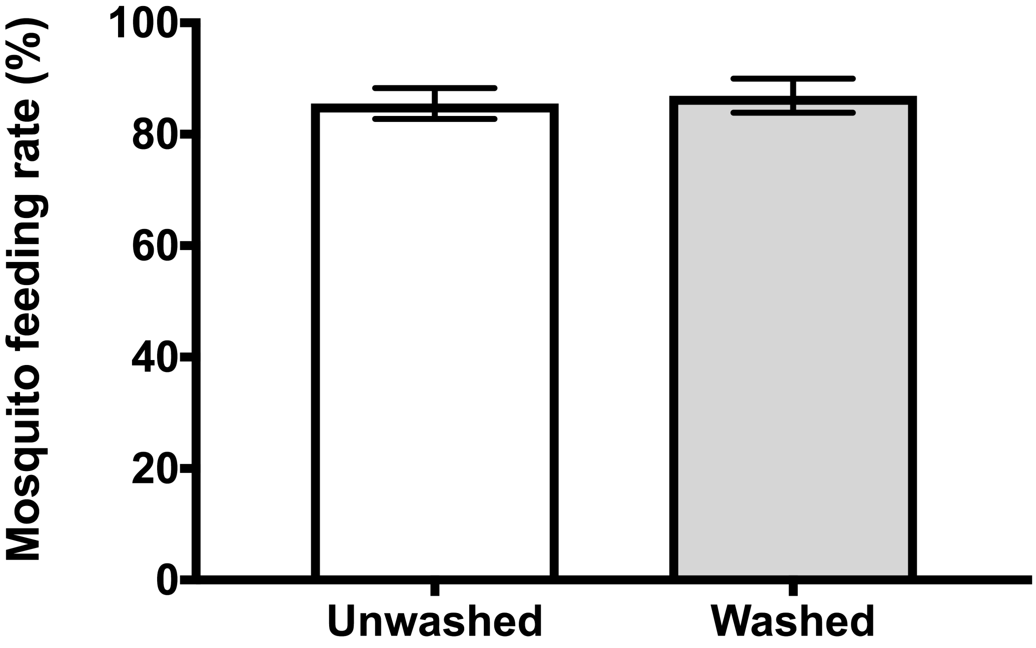

Figure S1. Effect of serum replacement on mosquito feeding rate of lab-colonized *An. stephensi.* Blood feeding rate was determined as the number of engorged mosquitoes/number of mosquitoes tested × 100. Whole blood (unwashed) and serum replaced-blood (washed) were used for feeding experiments. Data are represented as mean ± standard deviations from twenty independent experiments.
